# Surrogate endpoints ‐ clinical and analytical considerations for maximal utility in trial design, drug development and regulatory decision making

**DOI:** 10.1002/alz.083432

**Published:** 2025-01-09

**Authors:** Geert Molenberghs

**Affiliations:** ^1^ KU Leuven and Universiteit Hasselt, Leuven, Vlaams‐Brabant Belgium

## Abstract

An overview is given of surrogate marker evaluation, starting from the original definition of Prentice and his criteria, the estimation framework of Freedman, the meta‐analytic framework, and the evaluation of surrogate endpoints from a causal inference point of view.

Attention will be given, in particular, to evaluating tau‐PET as a reasonably likely surrogate in Alzheimer’s Disease. A meta‐analytic surrogate marker evaluation approach will be followed, for a continuous surrogate and a continuous true endpoint. Provisions are made to use both clinical trial data as well as natural history (real world) data. The statistical analysis consists of two steps: (1) a federated step where every site analyzes its own data, according to this protocol and the software provided; (2) a central step where the data hub processes the model outputs from the first step. The federated data analysis is a framework. As such, it can be applied based on a variety of choices made. Such choices pertain to patient population (e.g., early versus later stage), cognitive scale used (or, alternatively, sub‐scale or custom‐made item set), and particular tau PET measurement used (e.g., with variation over region of interest). A number of possible extensions are discussed too, as well as a single‐trial evaluation method to complement the federated meta‐analysis.

